# Uniting sex and eukaryote origins in an emerging oxygenic world

**DOI:** 10.1186/1745-6150-5-53

**Published:** 2010-08-23

**Authors:** Jeferson Gross, Debashish Bhattacharya

**Affiliations:** 1 Department of Ecology, Evolution and Natural Resources, and Institute of Marine and Coastal Sciences, Rutgers, The State University of New Jersey, Foran Hall 102, New Brunswick, NJ 08901, USA

## Abstract

**Background:**

Theories about eukaryote origins (eukaryogenesis) need to provide unified explanations for the emergence of diverse complex features that define this lineage. Models that propose a prokaryote-to-eukaryote transition are gridlocked between the opposing "phagocytosis first" and "mitochondria as seed" paradigms, neither of which fully explain the origins of eukaryote cell complexity. Sex (outcrossing with meiosis) is an example of an elaborate trait not yet satisfactorily addressed in theories about eukaryogenesis. The ancestral nature of meiosis and its dependence on eukaryote cell biology suggest that the emergence of sex and eukaryogenesis were simultaneous and synergic and may be explained by a common selective pressure.

**Presentation of the hypothesis:**

We propose that a local rise in oxygen levels, due to cyanobacterial photosynthesis in ancient Archean microenvironments, was highly toxic to the surrounding biota. This selective pressure drove the transformation of an archaeal (archaebacterial) lineage into the first eukaryotes. Key is that oxygen might have acted in synergy with environmental stresses such as ultraviolet (UV) radiation and/or desiccation that resulted in the accumulation of reactive oxygen species (ROS). The emergence of eukaryote features such as the endomembrane system and acquisition of the mitochondrion are posited as strategies to cope with a metabolic crisis in the cell plasma membrane and the accumulation of ROS, respectively. Selective pressure for efficient repair of ROS/UV-damaged DNA drove the evolution of sex, which required cell-cell fusions, cytoskeleton-mediated chromosome movement, and emergence of the nuclear envelope. Our model implies that evolution of sex and eukaryogenesis were inseparable processes.

**Testing the hypothesis:**

Several types of data can be used to test our hypothesis. These include paleontological predictions, simulation of ancient oxygenic microenvironments, and cell biological experiments with Archaea exposed to ROS and UV stresses. Studies of archaeal conjugation, prokaryotic DNA recombination, and the universality of nuclear-mediated meiotic activities might corroborate the hypothesis that sex and the nucleus evolved to support DNA repair.

**Implications of the hypothesis:**

Oxygen tolerance emerges as an important principle to investigate eukaryogenesis. The evolution of eukaryotic complexity might be best understood as a synergic process between key evolutionary innovations, of which meiosis (sex) played a central role.

**Reviewers:**

This manuscript was reviewed by Eugene V. Koonin, Anthony M. Poole, and Gáspár Jékely.

## Background

The birth of eukaryotes was a milestone in the evolution of life on our planet, yet the initial stages in this process remain shrouded in mystery. Even the most widely accepted notion that eukaryotes originated from prokaryotes is problematic because traits unique to eukaryotes, such as the nucleus, endomembrane system, cytoskeleton, and mitosis, are found in all taxa with no intermediate stages left as signposts of their evolution [1-3]. This results in the "chicken-and-egg" dilemma when attempting to explain the origin of eukaryote complexity from prokaryote ancestors. The best example of this conundrum is the acquisition of the alphaproteobacterium-derived precursor of mitochondria. Traditional hypotheses based on the "phagocytosis first" view posit the existence of a mitochondrion-less ancestor of eukaryotes (e.g., an "archezoan") that engulfed and retained the alphaproteobacterium *via *endosymbiosis [1-4]. The search for amitochondriate eukaryotes has thus far proved fruitless and the bulk of data suggest all living eukaryotes (whether currently housing this organelle or a derived version [e.g., hydrogenosome]) once shared a mitochondrion-containing ancestor [1]. Moreover phagocytosis is a derived property of the endomembrane system and cytoskeleton, both of which are highly ATP-needy [5], suggesting that the energetic output of aerobic mitochondria might have been required for the evolution of phagocytosis. The central importance of the mitochondrion to eukaryogenesis is recognized by alternative hypotheses that posit the acquisition of the mitochondrial forerunner by an archaeon host as the founding event of eukaryotic evolution (e.g., the hydrogen hypothesis) [1,2,6]. This "mitochondrion as seed" perspective fails however to satisfactorily explain how the mitochondrial forerunner gained entry into the host cell in the absence of phagocytosis and how the evolution of eukaryotic complexity evolved after this critical event [2,3].

The origin of sex is another shadowy avenue in eukaryote evolution. Many lines of evidence demonstrate that sex is beneficial for extant eukaryotes by creating genetic variability, masking or eliminating deleterious mutations, and assisting DNA repair processes [7-9]. Meiotic genes are however present in all major eukaryotic clades, suggesting that sex is ancient and predates diversification of extant eukaryotes [9]. In addition, sex as outcrossing can only occur if distinct, complex cellular activities, such as syngamy (i.e., fusion of gametes), karyogamy (i.e., fusion of nuclei), and meiosis, act in concert [10-12]. These processes depend inextricably on the dynamics of the cytoskeleton, endomembrane system, and nuclear envelope, on the structure of chromosomes, and are embedded in the complex eukaryote cell cycle [10-13]. Here we posit that such a global involvement of eukaryote cell biology in the process of sex and its early appearance in eukaryotes reflect the fact that the evolution of sex and the emergence of the eukaryote traits were synergic processes. These might have occurred in a population of archaeal cells as response to a unique combination of stressful conditions associated with the rise of oxygenic microenvironments on Earth.

## Presentation of the hypothesis

### Were eukaryotes forged by an oxygen crisis?

The paleontological and biomarker record place early vestiges of eukaryotes around 2.7 - 1.7 billion years ago (Gya) [14-16]. This suggests that eukaryotic origins may correlate with a major transition in Earth's geochemistry; i.e., the global accumulation of cyanobacterial-derived oxygen (O_2_) [15]. The large anomalous mass-independent fractionations of sulfur isotopes, produced by photoreactions mediated by ultraviolet (UV) light during the Archean, abruptly disappear by 2.4 Gya [15,17]. This indicates that O_2 _had accumulated in the atmosphere and formed an ozone-like photoscreen [15,17]. Mounting data, however, suggest that O_2 _had already started to spread through local shallow water environments during the Archean - Paleoproterozoic boundary (about 2.6 - 2.4 Gya) [17,18]. These observations point to a period in history in which early O_2 _proliferation likely occurred under an ozone-less atmosphere. During this exceptional circumstance, it is conceivable that deep penetrating UV radiation may have photo-activated a considerable fraction of the oxygenic pools into reactive oxygen species (ROS), such as the superoxide (O_2_^-^) ion and hydrogen peroxide (H_2_O_2_) [19-22]. In prevalently ferrous rich aquatic systems [17,18], these could have been further transformed *via *Fenton chemistry into highly reactive hydroxyl radicals [21]. Abundant production of ROS may have also occurred inside the cells. A key notion explored in this essay is that molecular oxygen (i.e., O_2_) alone is only a mild toxic agent and therefore O_2 _might have acted in concert (i.e., in synergism) with environmental stressors such as UV radiation and/or desiccation. Both of which are common in surface environments and lead to massive activation of intracellular O_2 _into ROS [23-25]. Taken together, these observations suggest that life forms faced a new challenge to survival when highly oxidative microenvironments, permeated by UV radiation, first emerged as a result of cyanobacterial photosynthesis.

### Rescuing the metabolic activities of the archaeal plasma membrane

The nuclear envelope (NE) is traditionally championed as the defining feature of eukaryotic cells. Despite this deep-rooted view, modern cell biology demonstrates that the NE is morphogenetically derived from the endoplasmic reticulum (ER) [2,26]. The ER is also the driver of endomembrane system biogenesis [27,28]. Therefore, does the function and evolution of the ER provide clues to early events in eukaryogenesis? Among many activities, the ER is the site of post- and co-translational protein targeting, core glycosylation of proteins, and biosynthesis of the majority of cell lipids and glycerophospholipids [27-30]. These processes lie at the heart of biological functions performed by the prokaryotic plasma membrane (the inner membrane in Gram-negative bacteria) [29-32]. There is also a topological correlation. Signal recognition particles (SRPs) and ribosomes bind to Sec translocases at the cytosolic face of the eukaryotic ER and the prokaryotic plasma membrane, respectively, and nascent proteins are co-translationally directed to the opposite side [29,30,33]. Notably, the core glycosylation system and subunits of the eukaryotic Sec, SRP, and ribosomal machineries are phylogenetically more closely related to archaeal counterparts than to homologs in Eubacteria [29-31,34,35]. Taken together these observations are consistent with three hypotheses: 1) the eukaryotic ER represents the remnants of the plasma membrane of ancestral Archaea; therefore, 2) the process of eukaryogenesis might best be understood in light of phylogenetic hypotheses that propose an Archaea-to-eukaryote transformation [34,35]; and finally 3) the origin of the ER might reflect a migration of the plasma membrane metabolic activities towards the cell interior. We suggest that the plasma membrane-to-ER metabolic shift was in fact the reason for the emergence of the endomembrane system. Might this have occurred as a response to detrimental environmental factors associated with the incipient raise of O_2_?

**Figure 1:**
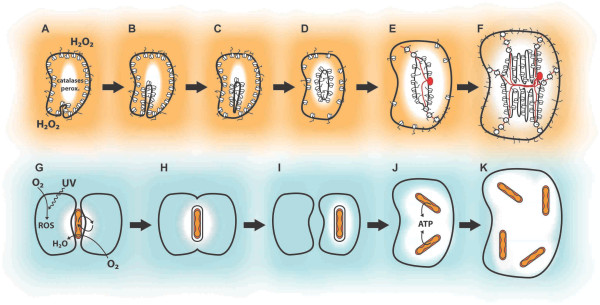
**Evolution of the endomembrane system, mitochondrion, and eukaryote cell size**. A - F. Model for evolution of the endomembrane system in response to imbalances in plasma membrane activities. Archaeal cells, containing co-translationally active ribosomes, are exposed to an environmental stressor (here exemplified by external H_2_O_2_, orange background, although UV radiation and/or desiccation may provide additional sources of stress). The plasma membrane was particularly affected by this external injury. As a result of the peripheral damage, a vesicle carrying molecular components (e.g., ribosomes) pinched off from the plasma membrane and accumulated in the inner cell, giving rise to the proto-ER. H_2_O_2 _that infiltrated the cell was cleared by enzymes (e.g., catalases and peroxidases). This generated a protected intracellular zone (white) that allowed proliferation of the proto-ER and associated ribosomes, while H_2_O_2_-damaged co-translational targeting gradually disappeared from the plasma membrane. Vesicular traffic, scaffolded by the incipient cytoskeleton (microtubule-organizing center and microtubules in red), emerged as an exocytic avenue to target ER-synthesized proteins to the plasma membrane (E and F). G - K. Putative model for early events in mitochondrial evolution. In a biofilm, archaeal and alphaprotobacterial cells are juxtaposed in a syntrophic association (arrows). Fusogenic and membrane remodeling activities necessary for cell-cell fusions during archaeal mating allowed the capture and retention of the alphaproteobacterium precursor of mitochondria. Environmental O_2 _(blue background) penetrates the cells and is photo-activated to ROS by UV. Alphaproteobacterial aerobic respiration clears the intracellular O_2 _(white zones). Intracellular mitochondria propagate and deliver ATP to the cytoplasm (J and H). Increase in cell size (E, F and J, K) emerges as a crucial eukaryotic strategy to counterbalance the influx of oxygenic species.

It is possible that the molecular activities at the archaeal plasma membrane were directly injured by environmental ROS and/or UV, both known to affect plasma membrane composition [36-39], and to deplete molecular factors of the translation machinery [40-42] and protein targeting [43]. In addition, it is possible that exposure to cycles of dehydration/rehydration during desiccation could critically affect plasma membrane integrity [23,44]. In contrast, the interior of the archaeal cell might have been protected from this type of peripheral damage. The proto-ER might therefore have been selected to rescue the metabolic activities of the plasma membrane in the safe interior milieu of the cell. This possibility makes sense under the hypothesis of environmental ROS exposure. Current data regarding cellular ROS detoxification demonstrate that the plasma membrane acts as a semi-permeable barrier that constrains diffusion rates of O_2 _and H_2_O_2 _(O_2_^- ^is efficiently halted by lipid bilayers), allowing the turnover of detoxifying enzymes, such as catalases, peroxidases, and terminal oxidases to counterbalance the influx of oxygen species (Figure 1A-F). The result is a steep extracellular/intracellular gradient of O_2 _and H_2_O_2 _concentrations [45,46]. If we assume that the proto-eukaryote encoded a set of ROS detoxifying enzymes, increase in cell size may have maximized the clearance of environmental oxygen species (including the clearance of O_2 _by the mitochondrion, see below) (Figure 1A-K) [46]. This also implies that metabolic activities housed in the proto-ER were shielded by cytoplasmic antioxidants and could proliferate in this compartment, whereas protein targeting in the plasma membrane was directly exposed to environmental injury and eventually went extinct from the periphery of the cell (Figure 1A-F). Whereas the protein targeting and metabolic activities proliferated in the proto-ER, they were under negative selection in the plasma membrane and eventually were lost from the periphery of the cell. Such a plasma membrane-to-ER shift however required that vesicular traffic evolved in parallel as an alternative avenue to load the plasma membrane with ER-derived proteins and metabolites (Figure 1E, F). This consolidated the ER as the metabolic factory of the cell and the secretory pathway as the driver of endomembrane system biogenesis [27,28,33]. But how did the first ER originate?

Proponents of "phagocytosis first" hypotheses suggest that the endomembrane system emerged as an organized endocytic pathway that eventually differentiated into the proto-ER [2,47]. This idea however cannot readily account for a shift in the plasma membrane-to-ER metabolic activities. In addition, it contradicts the actual flow of biogenetic information through the endomembrane system that proceeds from the ER to the plasma membrane (i.e., an exocytic pathway) [27,28,33]. Empirical observations in *Escherichia coli *indicate that endovesicles can directly accumulate as a result of overexpression or depletion of inner membrane proteins and thereby might provide an alternative and more plausible scenario for proto-ER emergence [48,49]. In particular, depletion of the SRP and the SecE subunit from the inner membrane protein translocon results in the accumulation of endomembranes that resemble the ER structure, including the co-localization of SRP receptors and ribosomal particles at the cytosolic leaflet of the new formed vesicles [48]. These observations support the idea that endomembranes co-segregating with key molecular machines (e.g., Sec translocons and ribosomes) could have spontaneously pinched off as a result of injuries to the plasma membrane of an ancestral Archaea and thereafter been selected to form the prototypic ER organelle.

### Mitochondria, the aerobic buffer of the eukaryotic cell

Was phagocytosis required for acquisition of the alphaproteobacterial endosymbiont precursor of mitochondria [1-4]? If phagocytosis is a derived property of the endomembrane system and cytoskeleton that consumes high amounts of ATP [5], the opposite is likely to have occurred; i.e., the aerobic mitochondrion was required for the evolution of phagocytosis. The presence in the ancestral Archaea of fusogenic and membrane deformation mechanisms in the context of a strong selective pressure might have been necessary and sufficient for acquisition of the mitochondrial forerunner. Consistent with this idea, phylogenetic data support the notion that the ancestral Archaea had homologs of eukaryotic Arp2/3 actin subunits, which presumably were capable of polymerization and promoting membrane deformation to eventually envelop an alphaproteobacterium [50]. In addition, the presence during conjugation in *Haloferax (Halobacterium) volcanii *of a network of partial fusions connecting diverse cells by protrusions of the plasma membrane referred to as cytoplasmic bridges illustrate that fusogenic and membrane deformation mechanisms exist in this domain of life [51,52]. Assuming that an analogous mating pathway was present in the ancestor of eukaryotes (discussed below) and that both alphaproteobacterial and archaeal populations were densely packed in a biofilm, it is conceivable that an alphaproteobacterium was "sandwiched" and enclosed between fusing archaeal cells (Figure 1G, H).

Whatever the mechanism, we suggest that capture of the alphaproteobacterial endosymbiont was crucial for survival in local oxidative environments that arose as a result of cyanobacterium-derived oxygen production. The idea that eukaryogenesis and alphaproteobacterial acquisition involved syntrophic symbiosis in the context of biofilms has been proposed more than once in the literature [2,6,53-55]. Here we recapitulate this notion by suggesting that the initial step of archaeal-alphaproteobacterial symbiosis might have been the formation of a defensive inter-species biofilm in which anaerobic archaeons were cross-protected by aerobic respiration provided by the alphaproteobacterium (i.e., O_2 _clearance, Figure 1G-K) [55,56]. Examples within the prokaryotic world are found in dental plaque biofilms [57] and in mixed cultures of aerotolerant bacteria with anaerobic sulfate reducing bacteria [58]. Later on, the capture of the aerobic alphaproteobacterium as an endosymbiont was a significant development that permitted efficient O_2 _scavenging inside the archaeal cell (e.g., as in the ciliate *Strombidium purpureum*) [56,59]. This step was crucial for survival because the proto-eukaryote was presumably exposed to substantial doses of UV and/or desiccation and therefore a large fraction of intracellular O_2 _would be activated into ROS (Figure 1G) [19,23-25]. Correspondingly, the endosymbiont was protected by the surrounding archaeal cytoplasm that contained ROS-detoxifying enzymes.

Endosymbiont-host cross-feeding might also have been possible. If both the archaeon and the alphaproteobacterium were heterotrophs, formation of syntrophic links (e.g., based on mono- and di-carboxylates, Figure 1G) between the symbiotic partners might have allowed dissimilation of archaeal redox equivalents and glycolitic end products (e.g., pyruvate) *via *alphaproteobacterial aerobic respiration [56,57,60]. This laid the foundation for extant cytosol-to-mitochondrion metabolic connections and eukaryotic aerobic metabolism, a condition *sine qua non *for long-term survival under oxidative stress. Strong selection to optimize incipient aerobic bioenergetics led the proto-eukaryote to progressively control activities of the alphaproteobacterium *via *establishment of host-encoded protein sorting systems in the endosymbiont [61]. This eventually converted the captured alphaproteobacterium into an ATP-producing organelle (Figure 1J), thereby providing the energetic surplus to support novel eukaryotic developments such as the endomembrane system and cytoskeleton.

### Did sex derive from archaeal mating?

**Figure 2:**
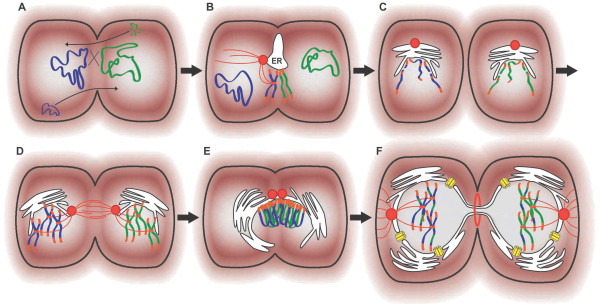
**A putative model for the evolution of meiosis from archaeal conjugation**. A. Ancestral archaeal conjugation (as described in *H. volcanii*) involving cell fusions, bidirectional flow of plasmids, and recombination between parental chromosomes (dark blue and green, respectively) [51,52]. B and C. Chromosome linearization permitted efficient pairing of homologues and resolution of crossovers [11,67]. Telomeres (orange) evolved to protect chromosome termini and to nucleate the pairing of homologues [11,67]. A centromere (orange region in the centre of chromosomes) served as a connection between sister chromatids and as an attachment site, *via *kinetochores, for the meiotic spindles [11,13,99]. This consisted of a network of microtubules (red fibers) radiating from a microtubule-organizing center (red circle) that guided chromosome movement [11,13,99]. The proto-ER progressively (B - F) differentiated into the NE [26] by wrapping segments of chromosomes to scaffold chromosome pairing (B - E) and to constrain diffusion of broken chromosome segments (C). D. Spindle-mediated movements approximate parental chromosomes during mating [10,12]. E. Incipient karyogamy mechanics evolved to fuse proto-NE segments associated with chromosomes to create a common membrane platform to assemble, *via *clustering of telomeres, the meiotic bouquet [11,13,67]. F. Cytokinesis based on an actomyosin contractile ring (red) facilitated splitting of the fusion partners (i.e., reductional meiotic division) [99]. NE enclosed the nuclear compartment when nuclear pores (yellow cylinders) evolved to ensure nucleo-cytoplasmic traffic of proteins and RNA [26].

Models for the origin of sex usually postulate its appearance in *bona fide *eukaryotes; i.e., nucleated cells capable of mitotic division [7,62]. In addition, current hypotheses for the emergence of sex usually suggest that crossover recombination between homologous chromosomes and ploidy reduction during meiosis evolved before outcrossing [47,62]. However, in modern eukaryotes outcrossing is only possible when features such as syngamy, karyogamy, and meiosis act in sequence [10-12]. If meiotic recombination and the nucleus evolved first, it is difficult to account for a later and simultaneous evolution of syngamy and karyogamy, since neither of these elaborate cell biological activities has a separate function to be selected for individually. Here we propose that outcrossing mediated by syngamy might have been an ancestral feature already present in the archaeal predecessor of eukaryotes. This idea is supported by the conjugational process observed in *H. volcanii *in which cycles of partial cell fusion and splitting allow reciprocal mobilization of plasmids and chromosomes, leading to ploidy variation (Figure 2A) [51,52]. This suggests an intriguing possibility; i.e., eukaryotic sex might have evolved from archaeal conjugation.

*Sulfolobus solfataricus *is an extant crenarchaeon in which conjugation involves tight cell-to-cell apposition and is mediated by biofilm formation in response to detrimental exposure to UV radiation [63-65]. Similarly, stressful conditions also trigger syngamy and sex in eukaryotic protists [66]. In *Sulfolobus*, UV induces pyrimidine dimers that generate double strand breaks (DSBs) during DNA replication. These DSBs are the signal for induction of conjugation-mediated recombination; i.e., DSBs generated by chemical agents also trigger conjugation [63,65]. In eukaryotic meiosis, DSBs are also formed after pre-meiotic DNA replication, however they are induced enzymatically by Spo11 [11,67], a component derived from the archaeal topoisomerase VIa subunit [9,67]. Notably, in taxonomically distantly related eukaryotes such as yeast and the nematode *Caenorhabditis elegans*, requirement of Spo11 can be partially bypassed by other agents that induce DSBs or single strand damage [68,69]. These observations indicate that DNA lesions expressed during replication, usually in the form of DSBs, represent a central and ancestral feature of both eukaryotic sex and conjugation in *Sulfolobus*. In eukaryotes, these DSBs are repaired during meiosis by homologous recombination between the two parental chromosomes [11,67-69]. This reaction is catalyzed by a machine whose central component, Dmc1 is evolutionarily related to archaeal RecA ([70]; Gross and Bhattacharya, unpublished data). In *Sulfolobus *and diverse archaeons DSBs are also presumably repaired by RecA-mediated homologous recombination that uses as intact templates genetic material mobilized during conjugation [63,65,71,72]. This RecA pathway for DSB repair involves a complex formed by Rad50-Mre11-HerA that is often encoded in the same operon in Archaea [70,73]. This is of interest because homologs of the HerA ATPase are implicated in DNA pumping during both binary division and conjugation in many prokaryotes, whereas Mre11 and Rad50 are core components of both the meiotic and mitotic recombination machineries [11,70,73]. These parallels between eukaryotic meiosis and archaeal conjugation not only strengthen the idea that the former evolved from the latter, but that both serve the same purpose, repair of DNA damage, in particular DSBs.

The analogies and interpretations above in part recapitulate ideas already elaborated in the traditional DNA repair hypothesis for the evolution of sex [8,66]. Here we advance these notions by proposing a scenario in which sex evolved to cope with the pervasive genotoxicity produced by ROS and UV in the harsh conditions that accompanied incipient O_2 _emergence on the surface of the Earth. If the ancestral state of pre-eukaryotic sex resembled the conjugational process observed in *H. volcanii*, it is conceivable that cell-cell fusions allowed the formation of diploid/polyploid states more resistant to deleterious mutations. In addition inter-cell mobilization of large chromosome segments by conjugative plasmids allowed DNA repair by homologous recombination (Figure 2A) [51,52,72]. We propose that such bidirectional genetic flow that was used for DNA repair, over time, evolved into meiosis (Figure 2B-F); i.e., a highly organized system to bring two parental DNAs in close proximity, to use whole choromosomes in a homologous recombination reaction, and to relocate the repaired chromosomes back to the donor cells while splitting the fusion partners (i.e., the meiotic reductional division). Here we posit that selection for the evolution of meiosis in addition served as a major driving force for a global restructuring of the archaeal cell (summarized in Figure 2). This occurred because meiosis required innovations such as linearization of chromosomes, emergence of telomeres, centromeres, the meiotic spindle, the ER-derived nuclear envelope, nuclear congression, karyogamy, meiotic bouquet, and cytokinesis mediated by an actomyosin contractile ring (for a discussion on the fundamental roles of these eukaryotic features in supporting sex and meiosis see next section and references [11,13,67]). Because the cytological dynamics of parental cell splitting during meiosis is essentially similar to non-meiotic binary division [13], it is conceivable that the incipient meiotic cell cycle was in parallel co-opted for mitosis. All these developments are seen as a result of strong selection to withstand long term ROS/UV genotoxicity, particularly in the form of DNA DSBs. However, over time DSBs were deliberately initiated by Spo11 catalysis after pre-meiotic replication [11,67-69]. This might imply that the byproduct of crossovers during homologous recombination, the genetic shuffling between parental chromosomes [8,11], might have been under selective pressure, suggesting that the generation of genetic diversity likely played a fundamental role in the evolution of primitive eukaryotes. The notable outcome of these complex developments driving the evolution of sex is however the emergence of the most archetypal of eukaryotic organelles.

### The nucleus, an arena for meiosis

The reason for the origin of the nucleus is still enigmatic. Although many different models have proposed a singular selective force [47,74-76], it is possible that diverse factors could have synergistically contributed to the evolution of the nuclear envelope. Our hypothesis provides a new perspective on nuclear origins. For meiotic recombination to occur chromosomes from two different parental cells must converge to a common cellular region, find the corresponding homologous chromosome, and pair in perfect homologous juxtaposition [11,13]. Given the dimensions of the cell, this remarkably complex task is unlikely to occur solely *via *random encounters resulting from the free diffusion of chromosomes [11]. We propose that the evolution of the nucleus was a requirement to significantly enhance the kinetics of homologous chromosome pairing (Figure 2). Following syngamy, the nucleus serves as a transport capsule to conjunctly move the whole set of chromosomes of one mating cell into close proximity of the partner nucleus (i.e., nuclear congression) [10,12]. Once in physical contact, the two nuclei fuse and constrain the two sets of parental chromosomes into a portion of the cell delimited by the NE [10]. Physical encounters between homologous chromosomes that follow karyogamy are orchestrated from the NE by a process that involves migration of chromosome telomeres towards a common convergent point in the NE in close contact with a microtubule-organizing center [11,13,67]. The meiotic bouquet, resulting from this collective attachment of telomeres to the NE, ensures a rough linear juxtaposition of the chromosome arms (Figure 2E). The detailed assessment of homology within the meiotic bouquet is also contributed by the nucleus *via *cytoskeleton-mediated nuclear oscillations. These movements are conveyed to the meiotic bouquet to increase the rates of encounters between homologous regions in the pairing chromosomes, while destabilizing weak non-homologous interactions [11,67]. Finally, stable chromosome pairing between homologues is accomplished by meiotic recombination. All of these nuclear-mediated activities are widely conserved among eukaryotes [11,13], in accordance to the notion that the nucleus originated to scaffold the process of meiosis.

In addition to meiotic recombination, the proto-nuclear compartment might also have been crucial to non-meiotic processes of DNA repair. DSBs resulting from ionizing radiation are efficiently repaired in radiotolerant bacteria such as *Deinococcus *species [24,77]. Underlying the extreme radiotolerance of these prokaryotes is a high level of nucleoid compaction, which constrains the dispersal by diffusion of broken chromosome fragments that are produced by radiation. This process maintains the original chromosome contiguity and greatly enhances the kinetics of chromosome reconstruction by homologous recombination and in particular, non-homologous end joining [77,78]. We propose that in proto-eukaryotes, nuclear confinement of the genome and its compaction by histones organized in nucleosomes served the same purpose; i.e., to restrict dispersal of chromosome fragments (Figure 2C) and to concentrate and spatially organize the molecular machines required for chromosome repair. To overcome the genotoxic load, the nucleus emerged therefore as an organelle dedicated to a diversity of DNA repair processes. Interestingly, the planctomycete *Gemmata obscuriglobus *contains a highly compacted nucleoid encapsulated in a double-membrane compartment analogous to the eukaryotic nucleus. This taxon has recently been demonstrated to be highly resistant to ionizing radiation, suggesting a correlation between nucleoid compartmentalization and high turnover of DNA DSB repair [78].

### Signatures of aerobic eukaryogenesis

The heterogeneous phylogenetic affiliation of the eukaryotic proteome provides a conundrum in traditional theories of eukaryogenesis [1,2,34]. We propose that the make-up of the eukaryotic proteome reflects two major selective forces that acted during eukaryogenesis to reformat the archaeal proteome *via *horizontal gene transfer (HGT), endosymbiotic gene transfer (EGT), and *de novo *evolution of a plethora of new proteins. These are: 1) the emergence of diverse new cellular functions and, 2) the conversion of archaeal metabolism to aerobiosis. These adaptations demanded a wholesale redesigning of metabolic flow and proteins that have different substrate affinities, allosteric regulation, redox sensitivity, and new enzymatic activities [79-81]. This resulted in a phylogenetic profile that is characterized by a large number of eukaryotic specific proteins [2,80] and a substantial fraction of eukaryotic operational proteins (e.g., metabolic enzymes) that have a eubacterial rather than archaeal provenance [1,2,34]. In contrast, informational proteins (e.g., those involved in gene expression, transcription, and translation) were less affected by replacements *via *HGT or EGT because they supported fundamental infrastructural functions in the newly evolved eukaryotic cell, and were therefore vertically inherited from the archaeal ancestor [1,2,34].

Aerobic eukaryogenesis is also reflected in two major innovations: 1) The emergence of peroxisomes, which were ER-derived compartments to house oxygen-using reactions that generate peroxides, quenched *in situ *by catalases [82] and, 2) the biosynthesis of sterols, which is strictly dependent on O_2 _(e.g., in yeast 12 O_2 _are used per 1 ergosterol that is synthesized) [83-85]. Sterols are critical for eukaryotes and lineages that cannot produce these compounds must obtain them from the environment [83-85] (with the notable exception of *Tetrahymena pyriformis *that synthesizes the sterol surrogate tetrahymanol [83]). This observation has been confirmed in yeast using genetic screens, whereby mutations that affect the critical initial steps of sterol biosynthesis are lethal [86]. It is conceivable that the catabolic consumption of O_2 _during sterol biosynthesis might have been a detoxification strategy for the proto-eukaryote. In addition, synthesized sterols might have provided further protection, serving as barriers to constrain the influx of O_2 _and H_2_O_2 _across the plasma membrane [46,83]. In this regard, diverse viable mutants in yeast impaired in the late steps of ergosterol biosynthesis show enhanced sensitivity to oxidative stress [46,87]. This might be explained by the observation that in wild-type yeast adaptation to H_2_O_2 _stress involves the redistribution of sterols in the membrane forming sterol-rich microdomains, which may restructure the plasma membrane, rendering it less permeable to H_2_O_2 _[88]. Not surprisingly the phospholipid/sterol ratio in the eukaryote plasma membrane can range from 0.5 - 1.0 [89]. Such an enrichment of sterols likely had a major impact on the lipid composition of the proto-eukaryote. The plasma membrane of Archaea is based on glycerophospholipids comprised of ether linked isoprenoid chains [2]. Sterols are also made of isoprenoids [84]. Therefore it is conceivable that the isoprenoid pool was largely consumed by the biosynthesis of sterols, leaving the cell in short supply of isoprenoid-based glycerophospholipids. This constrained the proto-eukaryote to adopt in their membranes the alternative eubacterial type of glycerophospholipids, based on ester bound fatty acids. This combination of sterols, eubacterial glycerophospholipids, and diverse lipids (e.g., sphingolipids) allowed high malleability of the endomembrane system, which is based on the presence of local domains enriched in particular lipid compositions [89]. Sterols are key modulators of such lipid rafts [84,88,89], therefore their emergence might have been a fundamental condition for the evolution of the endomembrane system and hence eukaryogenesis. It is noteworthy that the planctomycete *Gemmata obscuriglobus*, which is one of the rare prokaryotes known to synthesize sterols [84], has a malleable endomembrane system capable of endocytosis [90].

## Testing the hypothesis

Several aspects of our hypothesis can be tested using either direct or indirect means. Our central idea that eukaryotes evolved in the vicinity of oxygen-producing cyanobacteria in UV-permeated zones implies that the earliest fossil record of eukaryotes should be associated with paleontological sites that were once exposed on the Earth's surface. This idea is consistent with the recent description of potential eukaryote microfossils in 3.2 Gya old geological deposits corresponding to Archean shallow water marine environments [91]. The early appearance of eukaryotes is therefore constrained in a time span that extends from the emergence of cyanobacterial oxygenic photosynthesis (still an open issue [92]) to about 2.4 Gya, when an ozone layer likely was in place and shielded our planet from harmful UV-C radiation [15,17]. In addition, the fundamental role of oxygen-derived sterols in the evolution of the endomembrane system, as predicted by our hypothesis, suggests that sterol-related biomarkers might be found in association with the earliest vestiges of eukaryotes in the fossil record. Such ancient oxygenated and UV permeated environments can be simulated in controlled laboratory conditions and its impact on production of ROS measured quantitatively. The feasibility of similar UV-mediated photo-reactions in the presence of trace levels of O_2 _and water vapor was illustrated by simulations of conditions presumably found in Martian soil that led to substantial production of the O_2_^- ^ion [22]. It is therefore of high interest to test how microorganisms, especially archaeons, would be affected by constant exposure to UV/ROS. Little is known about the impact of UV and extracellular ROS on the plasma membrane components of Archaea. An interesting question that can be tested experimentally is whether endomembrane accumulation can be stimulated in Archaea as observed in *E. coli *by imbalances of plasma membrane components [48,49], or even by environmental stresses such as UV/ROS or desiccation.

We predict that the emerging concept in cell biology of radiotolerant prokaryotes, which posits a positive correlation between efficient DNA DSB repair and nucleoid compaction [24,77], will provide additional opportunities to test our idea that the nucleus emerged as an organelle to support general DNA repair. Planctomycetes are model systems to study the feasibility of this hypothesis. In particular, the nuclear-type compartment of *G. obscuriglobus *[78] might be a scaffold to assist DNA recombination, analogous to the hypothetical original function of the eukaryote nucleus in promoting diverse recombinational processes, including meiosis. Such a direct correlation between the nucleus and meiosis proposed here might imply that whenever meiosis is present in a given eukaryote, fundamental cytological features promoted by the nucleus, such as meiotic bouquet formation and cytoskeleton-mediated nuclear oscillations, should also be present [11,13]. We highlighted syngamy as the hypothetical ancestral feature of eukaryotic sex, possibly derived from its archaeal ancestor. Archaeal conjugation has thus far only been studied in *H. volcanii *and *Sulfolobus *species [51,52,63-65]. It would be of high interest to investigate in diverse archaeal taxa the role of cell-cell fusions and DNA repair processes mediated by conjugation. This might provide insights into the evolution of eukaryotic sex. In this regard it is important to isolate the molecular components involved in both syngamy in eukaryotes and in the formation of cytoplasmic bridges and/or cell-cell fusions in *Haloferax *or other archaeal taxa. A phylogenetic connection between core components involved in these processes might lend support to the idea of an evolutionary continuity between bacterial conjugation and eukaryotic sex. Generally, we predict that current and future molecular studies in extant archaeal taxa will strengthen the link between fundamental aspects of eukaryotic and archaeal cell biology (e.g., [93]). This might ultimately make it feasible in the future to achieve a more accurate picture of how Archaea were transformed into the first eukaryotes.

## Implications of the hypothesis

Here we propose that tolerance to oxygen may explain several aspects of eukaryote complexity including lineage origin. If this is true, then oxygen must have profoundly affected the development of eukaryote cell biology during evolution. This idea appears to be valid for the universal dependence of the endomembrane system on oxygen-derived sterols [83,84,89]. The fact that anaerobic eukaryotes, which cannot produce sterols, need to obtain this compound from food might imply that these organisms have been irreversibly marked by an ancestral aerobic life style [83,84]. Similarly, the universal biosynthesis of oxygen-sensitive Iron-Sulfur clusters in mitochondrion-like organelles [1], including the anaerobic mitosomes and hydrogenosomes, suggest a remnant function that was selected in the ancestral eukaryote to take place when oxygen is actively depleted by phosphorylative oxidation. The key role of oxygen species in signal transduction pathways controlling programmed cell death [94], cell differentiation processes [95], and adaptation to diverse stresses [66] might provide further examples of this paradigm. These may include the possible role of oxygen species in intermediate sexual processes [66,96]. In addition, oxygen also made possible the emergence of a plethora of new metabolic pathways that shaped eukaryote biochemistry [79]. Taken together, these observations serve to strengthen the argument that the highly exergonic respiratory chain enabled by oxygen was a thermodynamic requirement for the emergence of eukaryotic complexity [79,97]. Intriguing in this respect is the fact that the rise of oxygen levels in the late Proterozoic (about 0.85 - 0.54 Gya) [92] is also correlated with a major transition in eukaryote complexity involving sex; i.e., the evolution of multicellular body plans containing meiosis-specialized germ lines [98]. From oxygenic "Darwin ponds" located near cyanobacterial primary production, through the rise of multicellularity, the thermodynamics of oxygen chemistry might have profoundly influenced the trajectory of eukaryote evolution.

In light of our hypothesis that eukaryotes emerged from within Archaea it is important to pose the question why only a single archaeal group experienced this major evolutionary transition in the context of the broadly applicable selective pressure for oxygen tolerance. It is likely that the evolutionary success of the proto-eukaryotic stem lineage is explained not by single advances, but rather by the exquisitely rare synergistic establishment of different key innovations, such as acquisition of the mitochondrial forerunner, sterol biosynthesis, evolution of exocytic/endocytic pathways, the emergence of meiotic recombination (sex), and the nuclear envelope. Understanding the relative contribution of each of these novelties might provide a path for future investigations in eukaryogenesis.

## Competing interests

The authors declare that they have no competing interests.

## Authors' contributions

JG and DB conceived the hypothesis and wrote the manuscript. Both authors read and approved the final manuscript.

## Reviewers' comments

### Reviewer's report 1

*Eugene V. Koonin, The National Center for Biotechnology Information, National Library of Medicine*, *National Institutes of Health, Bethesda, Maryland, USA*.

The origin of the key features of the eukaryotic cell that are conserved in their complete, highly complex form in (almost) all eukaryotes and so are confidently mapped to the last common ancestor of all extant eukaryotes is a major enigma and a challenge to evolutionary biology. The only thing we seem to know beyond reasonable doubt about eukaryogenesis is that it involved a unique combination of events, including, in all likelihood, dramatic changes in the environment that the protoeukaryotes inhabited. Gross and Bhattacharya expand on this theme and suggest one key selective factor that could stand behind many if not all major eukaryotic innovations, the early oxygenation of the environment that, in the absence of the protective ozone layer, should have been highly damaging through the formation of reactive oxygen species (ROS).

### Author's response

*We appreciate these comments and the window it opens to underline a central point concerning oxygen toxicity as a driving force for eukaryogenesis. Under the best of circumstances molecular oxygen (O_2_) is only mildly toxic to some iron-sulfur clusters that are more exposed to the bulk solvent. Oxygen can only be widely damaging to biological structures when converted to ROS, especially in the form of the harmful hydroxyl radical. To our knowledge the major abiotic stresses commonly found in nature that produce ROS from O_2 _are UV and desiccation. It is important that these are usually overlapping stressful conditions that affect prokaryotes exposed to harsh environments on the Earth's surface. Therefore, our idea that eukaryotes were plagued by ROS implies that UV and/or desiccation were synergistic sources of stress whose respective contribution to overall biological damage can only be a matter of conjecture. In other worlds eukaryogenesis was driven by multiple associated factors*.

Indeed, there is strong evidence that such an incipient oxygenation was triggered by the evolving photosynthetic cyanobacteria, hence a strong selection pressure to evolve anti-ROS defense mechanisms. Gross and Bhattacharya discuss the evolution of these defenses. Of course, the most obvious one is the mitochondrial endosymbiont. We tend to think of mitochondria primary as "power plants" of eukaryotic cell but it is entirely plausible and indeed likely that the original pressure was for the more immediate role of the symbiotic alpha-proteobacteria in the defense against ROS. This is by no means a new idea (see Koonin, Aravind 2002. Cell Death Differ. 9: 394-404, and references therein) but Gross and Bhattacharya add specific, interesting ideas such as an initial stage of a symbiotic relationship within a biofilm preceding the actual endosymbiosis.

### Author's response

*This interesting observation made by the reviewer raises the opportunity to reinforce an important point in connection to our previous comment. The putative defense against ROS enabled by the mitochondrial forerunner was indirect. Our argument is that alphaproteobacteria/mitochondria could clear the O_2 _before it was transformed by UV penetrating the cell and/or desiccation into intracellular ROS. Some prokaryotes have inducible terminal oxidases that can also fulfill this task. However such a tolerance system is of short term efficacy because it is driven by consumption of NAD(P)H/ATP pools. Once the available redox equivalents are depleted, the cell collapses under persistent oxidative stress. The only way to cope with this condition is to couple oxygen consumption to regeneration of ATP and redox pools; i.e., to establish robust aerobic respiration. We propose this was a fundamental role of the alphaproteobacterium. This could only have been made possible if we assume a syntrophic link in which glycolytic end products of the Archaea were dissimilated by the aerobic respiration of the alphaproteobacterium in a way that glycolysis could constantly regenerate ATP and NAD(P)H pools. It is tempting to speculate that pyruvate was the key glycolytic end product that fed the mitochondrial forerunner because this is universally dissimilated by modern-day mitochondria, although any glycolytic derivative could have acted as the shuttle between the syntrophic partners*.

*Indeed both the idea of oxygen detoxification and syntrophy within biofilms are not new in the literature of eukaryogenesis and in our revised manuscript we acknowledge the original contributions*.

The second major innovation driven by the ROS stress is supposed to be the origin of the endomembrane system; here the specific driving force is thought to be protection of various enzymatic systems originally associated with the plasma membrane. This is quite a reasonable idea although, in contrast to the obvious relevance of mitochondria in ROS defense, the ideas on the involvement of the ER are further removed from the cause, and to me, have a certain flavor of a "just so story" (not that there are better ideas around...)

### Author's response

Although we respect the reviewer's opinion, given his comment and the objections posed by Anthony Poole (see below), we suggest that the logic underlying our model for the evolution of the endomembrane system might not yet have been clearly enunciated. We think that once these arguments are fully understood, the idea of a plasma membrane-to-ER metabolic shift, if not appealing, can at least be considered a plausible idea for discussion. Here we recapitulate the chain of arguments with the aim of clarifying our model:

*1) Why is it reasonable to argue that the ER derives from a prokaryotic plasma membrane? The ER is the site of co- and post-translational protein targeting, the synthesis of most of the membrane lipids, and glycosylation of proteins. All of these metabolic activities are core functions of the prokaryote plasma membrane. In addition to these obvious functional resemblances, there is a strict correlation between the ER and prokaryote plasma membrane with regard to the topological localization of ribosomes, Sec proteins, translocons, machineries for glycosylation of proteins and biosynthesis of lipids. The lumen of the ER is topologically analogous to the extracellular leaflet of the plasma membrane in bacteria, whereas the cytosolic exposed sides of both the ER and prokaryotic plasma membrane are also analogous with respect to topology. Taken together, all the topological and functional correlations described above strongly support the idea that the ER derives from of the plasma membrane, most likely by inward folding and budding*.

*2) Which prokaryotic group could be the best candidate to be the progenitor of the eukaryotic ER? We noted in our text the phylogenetic affiliation of the ER biosynthetic/topogenetic apparatus to Archaea. In addition, N-glycosylation of proteins is rare among Eubacteria, but widespread in Archaea. In the ER, the main subunit of the N-glycosylation enzymatic system, STT3, has an archaeal origin, and the carrier of glycosylated intermediates is dolichol-phosphate, that is also present in the membrane of Archaea. These observations led one of the reviewers of this paper to clearly state in a recent publication that "the topology of eukaryotic endomembranes originated via a single inward budding step from a precursor state similar to the one in modern archaebacteria" *[31]. *The idea that membrane topology is a solid hereditary unit emerged from the original studies of protein topogenesis and was crystallized by Günther Blobel's phrase "omnis membrane e membrane" (PNAS, 1980, 77(3): 1245-1247). It is as well championed by Cavalier Smith's concept of membrane heredity (Trends Plant Sci, 2000, 5(4):174-82). Protein translocons in membranes (and by extension protein sorting systems) act as replicators, since the assembly of a new translocon is catalyzed by another one pre-existing in the membrane (see reference *[61]*for a discussion). If we discount the unlikely possibility of en block HGTs, this intrinsic inheritable property of protein sorting systems, combined with the evident phylogenetic affiliation of the ER machinery to Archaea, strengthens the argument that the ER biosynthetic system was vertically transmitted from an ancestral Archaea that was the progenitor of eukaryotes*.

*3) Is it possible that the protein sorting system and associated metabolic functions were moved from the plasma membrane to the ER as a result of natural selection? Was there a problem with the plasma membrane? If we assume that points 1 and 2 are correct, then these two fundamental questions above come to mind. We can only speculate about selective forces that were acting at the cell periphery at the time of the birth of eukaryotes. However, since the plasma membrane is the interface between the cell and the external milieu, it is tempting to speculate that environmental factors were damaging the metabolic functions at the cell periphery, hence the plasma membrane-to-ER shift. Under this scenario the endomembrane system can be explained as an exocytic pathway because it evolved to export the fundamental components of the plasma membrane that can no longer be synthesized in this original compartment*.

*4) Is environmental ROS the best explanation for the plasma membrane-to-ER metabolic shift? In reality, we consider extracellular ROS as only part of the explanation. Here again the argument that oxidative stress comes in combination with UV and/or dissection is worthy of consideration. There exists compelling empirical evidence that ROS, UV, and desiccation cause specific damages to plasma membrane structures and inhibit the function of the protein sorting apparatus *[23,36-44]. *Our intention with the model developed in the text and in Figure *1A-F, *which focus on environmental ROS, was simply to illustrate how environmental damage explained the plasma membrane-to-ER metabolic shift. This could have been similarly illustrated by using UV or desiccation as putative sources of stress. We are aware that environmental ROS might not offer the complete explanation and that other still unknown factors contributed to the emergence of the ER from the plasma membrane. However, we hold to our idea that the plasma membrane-to-ER metabolic shift driven by negative selection at the cell periphery is important to understand eukaryogenesis, and thus merits serious consideration*.

The main focus of the paper is, of course, the origin of meiosis and sex, put into the same perspective. This part is strong as it puts together independent observations in archaeal and eukaryotic systems that connect the formation of DSBs with conjugation and meiosis, respectively. It is rather striking that in archaea spontaneously produced DSBs induce conjugation, as an entry into the RadA-mediated repair pathway, whereas in eukaryotes DSBs are introduced prior to meiosis, through a Spo11-mediated pathway. This connection is indeed very well compatible with the idea that archaeal conjugation is the evolutionary precursor of the eukaryotic meiosis.

Gross and Bhattacharya do not refer to a piece of evidence that seems central to the hypothesis on functional and evolutionary connections between DSB repair and cell division. In most archaea, the genes for the HerA ATPase and the NurA nuclease, the enzymes implicated in DNA pumping during both binary division and conjugation, appear to be encoded in the same operon with Mre11 and Rad50, the key enzymes of DSB repair (Iyer et al. 2004. Nucleic Acids Res. 32: 5260-5279). This evolutionarily conserved genomic association strongly suggests a tight functional connection between DSB repair and cell division/conjugation, and certainly, is compatible with the hypothesis that these two classes of DNA manipulation have been inseparable throughout evolution.

### Author's response

*We appreciate this information that has now been included in the revised version of the manuscript*.

Furthermore, biochemical study of the functions of all these archaeal proteins, including the complexes that they are predicted to form and more generally the mode of their cooperation, is the principal direction that has to be taken to test the hypothesis of the joint evolution of cell division (meiosis and probably also mitosis in eukaryotes); it seems to me that the current discussion of testing in Gross and Bhattacharya's article is somewhat sketchy and insufficiently concrete.

### Author's response

*In the testing section we refer more specifically to the machinery involved in syngamy in eukaryotes and to the mechanisms involved in cell-cell fusions in Archaea. If there is an evolutionary continuity between eukaryotic syngamy and archaeal cell-cell fusions the identification of the respective molecular machines should provide a fundamental correlation between these processes, most likely involving conservation of core molecular components. We agree with this criticism and have therefore restated the prediction in a more clear fashion. However we don't feel it is necessary to suggest a detailed experimental program. The analogies between DNA DSB-induced conjugation in Sulfolobus and eukaryotic meiosis discussed in the text already provide examples of how these two processes may correlate. We await in-depth molecular studies that may uncover more links between these two processes*.

The rest of the discussion in the article to me looks like a collection of somewhat far-fetched afterthoughts. In particular, the connection between chromatin compaction and radioresistance is tenuous at best. As far as I know, the most radioresistant organism currently known is Pyrococcus but there is no indication of any special chromatin compaction in this case. Conversely, Chlamydia with their very compact nucleoids, do not seem to show any special radiation resistance. Actually, in my opinion, the entire subject of compartmentalization in Planctomycetes-Verrucomicrobia-Chlamydia (however interesting in itself) is, at the very best, tangentially relevant to the subjects addressed in the article, so I think that omission of this part would actually tighten the argument (in the very least, the mention of Planctomycetes in the abstract in the same sentence with archaea is misleading). Furthermore, the suggestion that chromatin compaction, presumably needed to increase resistance to ROS and UV, was the driving force behind the evolution of the nucleus, is weak, in my opinion.

### Author's response

*We respect the fact that this reviewer favors a particular hypothesis on the origin of the nucleus (see next reviewer's comment). It is to be expected that such a controversial subject may divide the opinions of researchers in the field. However one of the most important ideas raised in our paper is that the evolution of meiotic recombination was the major driving force for the emergence of the nucleus in the proto-eukaryote. In the section "Nucleus, an arena for meiosis" we develop this idea and emphasize the essential role that the nucleus has in organizing the process of homology search and recombination during meiosis in all studied eukaryotes (see also Figure *2). *The fact that the nucleus has an intrinsic and universal role in the process of meiotic recombination cannot, in our opinion, be ascribed to chance or merely understood as a late evolutionary outcome. Furthermore, the argument that linear chromosomes, telomeres, centromeres, meiotic spindles, kinetochores, were pre-requirements for the evolution of meiosis has surfaced many times in the literature regarding the evolution of sex (i.e., from the perspective of the mitosis first hypotheses *[62,67]). *Here we simply invert the argument by suggesting that the evolution of these chromosomal structures was not a pre-adaptation, but rather, originally selected for meiosis. This connection between nuclear architecture, chromosome structure, and meiosis, seems to us to be key to understand the evolution of eukaryotes, and we believe that such correlations deserve to be seriously considered in eukaryogenesis*.

*The connection between radioresistance and chromatic compaction is a minor, though important, point of our model. Such a correlation was uncovered by recent research in radiotolerant bacteria, particularly Deinococcae (see *[24,77]). *To the best of our knowledge the nucleoid state of Pyrococcus, whether compact or not, has not been investigated with respect to cellular radioresistance. Nucleoid compaction is found exclusively in infectious extracellular forms of Chlamydia and presumably has a protective function (PNAS, 2004, 101(19): 7451-7456). Despite this function, it would be naïve for us to suggest that chromatin compaction and nuclear envelope in the proto-eukaryote merely served as protective wraps that shielded DNA against UV and ROS. Deinococcus extreme resistance, for example, does not rely much on shields or protective strategies, but rather depends on a robust post-damage response to reconstitute the chromosomes *[24,77]. *It is during this phase that a compact nucleiod is of extreme selective advantage because it can immobilize the broken DNA fragments relative to their original chromosomal position, thereby enhancing the kinetics of chromosome reconstitution by DNA repair enzymes. Furthermore, the scaffold afforded by a compact nucleiod is an ideal platform for concentration, assembly, and organization of DNA repair machines*.

*We brought up these essential ideas above in connection with the hypothesis of nuclear evolution being driven by meiosis, because concepts such as molecular crowding, spatial organization of DNA repair machines, and enhanced kinetics of DNA repair by restricted diffusion of substrates also apply to the function of the nucleus during meiotic recombination. There is a growing consensus among cell biologists that processes of DNA recombination involving homologous searching do not occur by free diffusion, but rather depend on spatial organization of specific repair foci in the cell (for a discussion see Nat Rev Microbiol, 2009, 7(10):748-55). In this regard it is notable that nuclear pore complexes are sites for repair of recalcitrant DNA DSBs, and that in many species such as yeast, the nuclear envelope has a fundamental role in the maintenance of telomeres. In addition, silencing of heterochromatic regions of chromosomes seems to be a conserved function of the nuclear periphery of eukaryotes. A plethora of studies have demonstrated that eukaryotic chromatin is not randomly dispersed within the nucleus and that nuclear architecture restricts chromosome movements creating territories and orchestrating the spatial organization of functional subdomains associated with chromosomes (for a discussion of the nuclear functions mentioned above see Nat Rev Mol Cell Biol, 2010, 11(5):317-28). Therefore we consider it plausible that spatial organization was the original function of the nucleus and that meiosis, which depends on highly organized physical constraints on chromosome movement, may have been the driving force behind the evolution of this compartment*.

*We prefer to retain the discussion involving Planctomycetes because this prokaryotic group represents a relevant model system to test ideas about the origin of eukaryotes. It is conceivable that Planctomycetes have independently recapitulated the evolution of an endomembrane system and membrane-bound compartments. It will be interesting to experimentally test in Gemmata obscuriglobus whether the existence of its nucleus-like compartment is explained by radioresistance, and it serves to restrict diffusion of chromosome fragments, to concentrate molecular machines, and to kinetically facilitate the process of DNA repair. This might not prove to be true, but will in any case test our model for evolution of the eukaryotic nucleus*.

In the least, an alternative and, in my opinion, more consistent scenario that derives the nucleus as a defense system against invading introns (Martin, Koonin 2006. Nature 440: 41-45; Moreira, Lopez-Garcia 2006. BioEssays 28: 525-533). More generally, it is naïve to attribute the emergence of a highly complex, multifunctional cellular machine like the nucleus to a single selective factor. Beyond doubt, there are multiple forces at play, and the best we can do, is to infer their relative contributions.

### Author's response

*We agree that a heuristic understanding of the evolution of the nucleus is a point to be debated and we acknowledge this idea in the new version of the manuscript. We now cite these and other publications that present alternative hypotheses for the origin of the nuclear compartment*.

To summarize, this review is not supposed to be strongly critical. I do believe that Gross and Bhattacharya propose a new (even if rooted in previous work) and viable scenario for the origin of meiosis (and, potentially, mitosis). Under this scenario, the ancient oxygenation of the environment created the pressure on the emerging eukaryote both to retain the pro-mitochondrial endosymbiont and to evolve efficient mechanisms of DSB repair that are tightly linked to DNA segregation mechanisms required for meiosis. Almost inevitably for this type of analysis, the discussion involves some overstretching as the authors try to explain as many eukaryotic innovations as possible within the framework of their model.

### Author's response

*We appreciated this reviewer's comments. The response to his comments helped enrich and further illustrate many key points of our hypothesis*.

### Reviewer's report 2

Anthony M. Poole, PhD, Department of Molecular Biology & Functional Genomics, Arrhenius Laboratories for Natural Sciences, Stockholm University, SE-106 91 Stockholm, Sweden

This manuscript considers a possible source of selection driving the origin of eukaryote cell architecture, and presents several interesting ideas. One major part of the manuscript is devoted to the impact of reactive oxygen species (ROS) on archaeal cells. The basic idea here seems to be similar to the ox-tox hypothesis (Andersson & Kurland 1999, Curr Opin Microbiol 2:535 - Note to authors: it might be fair to cite this paper), though in the present case the host is an archaeon, not a protoeukaryote.

### Author's response

*Although in the original version we acknowledged the contributions of Andersson & Kurland to the ox-tox model by citing their paper of 2000 *[56], *we agree with the reviewer that the publication of 1999 is more important because it corresponds to the original formulation of the hypothesis*.

The take on oxygen stress presented by Gross & Bhattacharya is however different from the ox-tox hypothesis in several respects. Significantly, they argue that the generation of ROS in the environment would have led to archaeal membrane damage, and that, in turn the eukaryotic ER is a remnant of the plasma membrane of the archaeal lineage from which eukaryotes are proposed to have evolved. The emergence of a proto-ER in this ancestral archaeal population would enable the interior of the cell to be protected from this stress.

### Author's response

*Again we emphasize that environmental ROS acting on the plasma membrane is only an illustration of a possible environmental stress that damaged the archaeal cell ancestor of eukaryotes. Other still unknown stress factors might have as well contributed (see our comments above to Eugene Koonin's review)*.

I particularly liked the point made in the section 'Signatures of aerobic eukaryogenesis' that sterol synthesis is oxygen dependent, and the following suggestion, that sterol synthesis could in itself serve as a detoxification strategy since synthesis of a single molecule of ergosterol in yeast consumes 12 O_2 _molecules. I would like to see this section elaborated upon, as the idea raises a lot of interesting questions and there may perhaps be data lurking in the literature that can be brought to bear on this hypothesis. For example, in the final section, the statement is made that anaerobic eukaryotes cannot produce sterols and need to obtain these from food. This fascinating point should be made earlier. It would also be very useful to provide references for this statement.

Returning to ergosterol synthesis in yeast, several questions come to mind. What happens to the sterol content of yeast kept in anaerobic conditions for extended periods? Are such yeast are more susceptible to ROS-related damage? Are there viable knockout yeast strains that cannot synthesise sterol, and to what degree are they sensitive to ROSs? Do the authors have any idea if there are published data relating to this?

### Author's response

*We were delighted to see that this referee appreciated our idea that sterols were crucial for eukaryogenesis and that this is still reflected by the essential requirement of sterols for survival in modern-day eukaryotes. This idea is supported the fact that yeast cannot grow under anaerobic conditions unless sterols are provided in the nutrient medium. We have now expanded the discussion on sterol requirement in eukaryotes and refer to empirical data that demonstrate the role of sterols in ROS defense in yeast*.

A weakness with the model as it currently presented is that it is not clear why all archaea did not adapt to this environmental stress by evolving additional membranes. There are plenty of extant archaea that exist in aerobic environments, yet as far as I am aware, their membranes are not eukaryote-like. To argue that this is a plausible source of selection for major membrane reorganisation in only *some *ancestral archaeal lineages therefore requires further explanation. One could ask the same of bacterial membrane architecture. What is the explanation for this environmental pressure so drastically impacting only *some *of the species present at the time, namely the ancestors of eukaryotes?

### Author's response

This is a fundamental point that merits discussion and we have done so in the final section "implications of the hypothesis" that highlights issues that need to be addressed in future studies of eukaryogenesis. The idea that a single putative group of Archaea developed eukaryotic features should not be taken as a weakness of our model. The fact that one can confidently ascribe all eukaryotic innovations to one single ancestor already implies that either only a particular group of primitive prokaryotes was driven to eukaryogenesis or that eukaryotes are a result of a single, successful "evolutionary experiment", whereas all other similar and parallel "evolutionary attempts" have gone extinct. Note that the same criticism can be leveled at any current model of eukaryotic origin. To provide an example of this with the "mitochondrial first" hypotheses, one may similarly pose the question why all eukaryotes trace their origin to a single prokaryotic host that acquired an endosymbiont, thereby triggering the transformation of host cell architecture?

*We suggest that the evolution of eukaryotes was a singularity because it involved a series of determinant innovations that conditioned the emergence of the remaining eukaryotic features. It was probably the synergism between different innovations (discussed in the text) that made the evolution of eukaryotes possible. A similar line of reasoning is used to explain the widely recognized single origin of the plastid. This event occurred hundreds of millions of years ago via the engulfment and transformation of a captured cyanobacterium by the putative ancestor of Plantae. Given the broad distribution of phagotrophy among unicells, why then are there not thousands of independent derivations of "Plantae"-like lineages on our planet? Again, because of rare and combinatorial events that favored the Plantae ancestor at the cost of its competitors, a single lineage rose to prominence and ultimately gave rise to all eukaryotic photosynthetic organelles (except of course for the special case of photosynthetic Paulinella species)*.

Regarding the supposed incompatibility of 'mitochondria-first' and 'phagocytosis-first' models, I agree that there is disagreement within the community, but I think the basis of the disagreement has no substance and is entirely imaginary. If both mitochondria and phagocytosis can be placed in LECA, there is really no need for a mitochondria-first model. Phagocytosis is not specifically tied to the defunct archezoa hypothesis or to a sister relationship between archaea and eukaryotes. If the hypothesis that eukaryotes evolved from within archaea is correct, phagocytosis simply evolves before engulfment of the mitochondrial ancestor. The alternative requires emergence of some alternative mechanism for entry (for which we currently have no evidence). This entry mechanism must only have occurred in (or affected) this specific archaeal lineage (which is the same as saying the eukaryote stem), but the mechanism was then lost and replaced by phagocytosis. So, starting from an archaeal ancestor, one either has one mechanism, still in action today (phagocytosis) or two (one which evolved and disappeared only in the direct archaeal lineage ancestral to modern eukaryotes, plus phagocytosis, which evolved prior to radiation of extant eukaryotes and is widespread among extant eukaryotes).

### Author's response

*The reviewer raises here an import point of debate. We do not favor the "phagocytosis first" hypothesis because phagocytosis, as a regulated system to engulf cells, represents a highly complex cell biological process. For example, it requires controlled mechanisms for sensing the presence of an external prey and transducing the signals into the capture event. This depends on the polymerization of actin filaments that mediate deformation of the plasma membrane around the prey *[50], *a highly complex and ATP spending activity *[5]. *Controlled fusogenic activities are necessary to seal the prey inside the cell, already implying the existence of a relatively highly developed endomembrane system. Important, once engulfed, the phagosome progressively maturates into a lysosome. This developmental route is embedded within the endocytic pathway that, by its turn, depends on membranes and molecular components derived through the canonical ER-Golgi route (Molecular cell biology, 2009, 10(9):623-635). In summary, when analyzed under a cell biological perspective, phagocytosis should be described as a highly derived trait of the endomembrane system. If phagocytosis was present in the LECA, we believe that it was most likely one of the last features added to the eukaryotic stem lineage. However the interpretation that the endomembrane system emanates from a rudimentary endocytic pathway that first evolved to engulf microparticles of food *[2], *seems to us more realistic and worthy of serious discussion, although this idea likewise contradicts the fact that the biogenesis and morphogenesis of the endomembrane system in based on an exocytic pathway*.

*With regard to the question of how the alphaproteobacterum gained entry to the archaeal ancestor of eukaryotes, it is logical to argue, as the reviewer did, that the mechanism should be a physiological process, such as phagocytosis. What we are however pointing out in our text is that the capture might have been merely accidental. It was necessary that the archaeal ancestor of eukaryotes had a relatively plastic membrane possessing fusogenic properties. This is conceivable if we assume that the progenitor of eukaryotes putatively had the ability to form cytoplasmic bridges and cell-cell fusions during conjugation, as observed in H. volcanii (vide Figure *1G*and *1H). *It is important to note that by developing the biosynthesis of sterols, as postulated in our model, the proto-eukaryote was equipped with special membrane flexibility and fusogenic properties. In addition, our model implies that the archaeal plasma membrane was unstable owing to environmental stress and that a primitive cytoskeleton and endomembrane system had already started to develop. In the context of biofilms, where cell-to-cell contacts are optimized, the above-mentioned plastic membrane properties might have facilitated the accidental entrapment and encapsulation of an alphaproteobacterial endosymbiont. Its retention inside the host cell however might be explained by the strong selective advantage that clearance of oxygen in the cytosol gave to the proto-eukaryote*.

On page 7, the authors present three 'key ideas': '1) the eukaryotic ER represents the remnants of the plasma membrane of ancestral Archaea; therefore, 2) the process of eukaryogenesis might best be understood in light of phylogenetic hypotheses that propose an Archaea-to-eukaryote transformation [34,35]; and finally 3) the origin of the ER might reflect a migration of the plasma membrane metabolic activities towards the cell interior.' I do not think the evidence in favour of 1) is sufficiently strong to state '1)...therefore, 2)' - it would seem more reasonable to make the 2) the starting assumption, and then follow with points 1) and 3), since both are suggestions lacking definitive evidence.

### Author's response

*We respect the reviewer's opinion, but we suggest that there are important existing data indicating that the ER derived from an archaeal plasma membrane. These are discussed in depth in our text and in our reply to Eugene Koonin's comments*.

The second major idea presented here is that sex evolved to cope with ROS-induced damage of DNA. The idea here is that cell-cell fusions led to increased ploidy, thereby buffering against deleterious mutations. This is an interesting idea, but again fails to explain the specificity of selection - why do only those archaea which evolve into eukaryotes develop this trait in response to a more globally-applied selection pressure. It is reasonable to suppose that other archaeal lineages would also be affected -- how are these lineages proposed to survive this 'oxygen crisis'?

### Author's response

*What we argue is that the ancestral Archaea was already fusing, and therefore the fusion of cells was not an innovation that affected a single lineage. In contrast, we propose the hypothesis that cell-to-cell fusions might be a more common feature even in modern-day archaeal conjugation. Our argument is that meiosis was a development of an existing primordial form of cell-cell conjugation. Although we briefly mention that increased ploidy would contribute to survival by buffering deleterious mutations, this is not the main argument. The central point in our model is that parental chromosomes when mixed in the same cell served as templates for a reciprocal repair reaction by homologous recombination*.

*How different lineages survive genotoxic injury is illustrated in our text by the example of Sulfolobus, which evolved bacterial conjugation that apparently serves to limit DNA DSBs *[63-65]. *Halophilic Archaea tend to be resistant to UV radiation and ROS by mechanisms that include homologous recombination, presumably driving genetic exchange in wild populations *(Saline Systems, *2005, 1:3). Integrative conjugative plasmids in Bacillus subtilis (PNAS, 2005, 102:12554-12559) Streptococcus thermophilus (J Bacteriol, 2009, 191(8):2764-75), and Vibrio cholerae (Nature, 2004, 427:72-74) have been demonstrated to be activated by DNA damage and cause widespread genetic recombination among cells. Competence for bacterial transformation (also considered a sexual process) has been associated with the expression of RecA in diverse prokaryotic groups and the state of competence is directly induced by DNA damage agents in Streptococcus pneumonia. In fact Bacillus subtilis is more resistant to UV/ROS genotoxicity by the uptake of DNA from the medium (Infect Genet Evol, 2008, 8:267-285). These examples illustrate that sexual processes triggered by DNA damage both in Archaea and Eubacteria seem to be more widespread than this reviewer may appreciate, and that globally-applied selection pressure caused by DNA damage led many times to a similar solution in distantly related taxa. Of course meiosis is a unique evolutionary innovation restricted to the eukaryote stem lineage. But here again we should consider the argument that the evolution of meiosis involved the implementation of diverse innovations and a special combination of events that currently are impossible to specify*.

As per the above discussion of membranes, increasing ploidy via cell fusion does not seem to be a widespread solution for limiting DNA-damage. Lineages that are frequently exposed to conditions of extreme DNA damage, such as the Deinococcaceae do exhibit polyploidy (Cox & Battista 2005, Nat Rev Microbiol 3:882), but repair is not facilitated by cell fusions -- as far as I know, no cases of syngamy have been reported in radioresistant lineages.

### Author's response

*It seems that there is an evolutionary trade-off between the energetic costs of maintaining multiple polyploid replicons within a cell and the acquisition by outcrossing of an external template for homologous recombination. Polyploid states are common in many prokaryotes during the exponential growth phase (PLoS One, 2006, 1:e92). In this case homologous recombination between sister chromosomes is favored. However during stationary phase, starvation prevails and multiple replicons are energetically costly. At this point conjugation or transformation is advantageous because it provides external information for DNA repair (Infect Genet Evol, 2008, 8:267-285). It might be the case that proto-eukaryotic cells also experienced such a trade-off during their evolution*.

*Another point is that sex might not have evolved solely to support DNA repair. The fusion of cells generating diploid/polyploidy states followed by recombination could promote selectable genetic diversity. In addition, sex is a means of propagation for selfish DNA elements. Integrative conjugative plasmids in prokaryotes act as "prophages" that excise from the genome under conditions of DNA damage and spread to other genomes through conjugation (PNAS, 2005, 102:12554-12559). The balance between these evolutionary forces might have contributed to a unique combination that led to the evolution of eukaryotic sex*.

*The connection between syngamy and radioresistance in the prokaryotic world should be investigated in more detail. Halophilic Archaea, such as H. volcanii, display UV and ROS resistance and prolific environmental recombination that is probably facilitated via conjugation *(Saline Systems, *2005, 1:3). In H. volcanii, cell-cell fusions and cytoplasmic bridges mediate the process of DNA recombination between parental chromosomes *[51,52].

Having said that, the discussion of conjugation in *Haloferax volcanii *and *Sulfolobus solfataricus *is certainly interesting, and the authors provide much food for thought here. The fascinating phenomenon of zygogenesis in *E. coli *(Gratia 2005, Microbiol 151:2947) is also interesting in this regard. Here it seems diploids can form, possibly via sites of membrane fusion, but I don't think there is evidence for recombination; rather, it seems that one chromosome is inactivated. These examples are perhaps all interesting in regard to the current proposal, but again, what I am missing is an explanation as to how come this solution was necessary in only a subset of the affected archaeal lineages (i.e. the archaeal ancestors of eukaryotes).

### Author's response

*The phenomenon of zygogenesis in E. coli is an interesting observation by this reviewer, noting that cell-cell fusions exist in the prokaryote world. Once again we reinforce the idea that cell-to-cell fusions were probably not a solution exclusive to the ancestor of eukaryotes, but might be a widespread feature even in modern-day Archaea. This idea merits testing. The fact that the progenitor of eukaryotes was already fusing was a starting point for the progressive evolution of organized homologous recombination reactions involving whole parental chromosomes; i.e., meiotic recombination*.

A more general point is whether rare catastrophic events are central to explanations of major changes in cell architecture. The very recent demonstration of an endocytosis-like mechanism for protein uptake in the planctomycete bacterium *Gemmata obscuriglobus *(Lonhienne et al. 2010, PNAS, PMID: 20566852) shows that an endomembrane system with vesicles and endocytosis has evolved more than once.

### Author's response

*We agree with the reviewer, however the evolution of this planctomycete might have included an "oxygen catastrophe". Radioresistance in G. obscuriglobus might be a by-product of tolerance to desiccation, a stress that is widely recognized to cause massive ROS damage to the cell *[24]. *This is why we believe it is important to study Planctomycetes to gain an understanding (however indirect) of fundamental aspects of eukaryogenesis. It is of note that G. obscuriglobus is a sterol producing bacterium *[84].

### Reviewer's report 3

Gáspár Jékely, Max Planck Institute for Developmental Biology, Tübingen, Germany.

This paper presents an interesting new hypothesis about the origin of eukaryotes. The authors work out a scenario in which accumulating oxygen and ROS plays a major driving factor during the rise of eukaryotes, including the origin of key cellular features such as the endomembrane system. There are many interesting ideas in the paper, it is cell biologically feasible, and it is well written.

One of my concerns is if the paleontological record can tell us anything about the role of oxygen as a causative agent in eukaryote origins, given the extremely broad possible time span for the event (the authors write 2.7-1.7 Gya). The evidence is convincing in the case of the Ediacaran fauna, but unfortunately we lack such evidence for eukaryotes.

### Author's response

*The oldest paleontological record is represented by sterol biomarkers of about 2.7 Gyr that provides evidence for the existence of a oxygen driven metabolic pathway. We agree with the reviewer that is difficult to use the fossil record to prove the influence of oxygen on eukaryogenesis. This idea however implies that remnants of primitive eukaryotes should be found in the Archean fossil record within the photic zone where oxygen was present*.

However, given the many ancestral oxygenic cell biological features of eukaryotes (peroxisomes, respiration etc.) it is clear that we have to consider an oxygenic world. But then, if oxygen was driving the changes globally and for a billion years, why was it only one lineage that developed internal membranes to prevent ROS damage?

### Author's response

*See our comments to similar concerns raised by Anthony Poole*.

More generally, one thing that this scenario doesn't provide is a unique event (cf. the origin of mitochondria or the origin of the first phagotrophic cell) that makes eukaryotes successful. It may be an error to single out one factor to explain the origin of eukaryotes. It is probably the same trap that 'phagocytosis only' and 'mitochondria only' scenarios can easily fall into. My feeling is that in future models we will have to understand the synergies between mitochondria and phagocytosis in an emerging oxygenic world.

### Author's response

*We fully embrace the idea that the complexity of eukaryotes is a result of synergistic evolution of multiple traits, and now we bring such a conclusion to the last section of our paper. Although we don't favor the notion that phagocytosis was the driver of initial eukaryogenesis, incipient endocytosis to acquire nutrients from environment can underlie the evolution of the endomembrane system. This idea deserves to be discussed in parallel to our "exocytosis first" model (which is conceptually similar to a model proposed by the reviewer himself *[31,33]).

The authors posit a fully developed archaebacterial cell as the ancestor of eukaryotes. They should point out that this is far from being a resolved issue. Archaebacteria are often shown as sisters to eukaryotes in sequence trees in which case their last common ancestor need not have been an archaebacterium.

### Author's response

*This is a controversial issue, although the association of eukaryotes to the crenarchaeal group has recently gained momentum *[35]. *As some traits putatively placed in the ancestor of eukaryotes seem to be proximal to what is observed in modern-day Archaea (e.g., the ER topology/functions as compared to modern archaeal plasma membrane *[31]) *we tend to favor the idea that eukaryotes split from the archaeal supergroup*.

The discussion about the origin of mitochondria and phagocytosis is too speculative and follows an unfortunate trend in the literature. One can just as well say that the archaebacterial proto-cytoskeleton with Arp2/3 and the ability to fuse already worked as a primitive phagocytic machinery. At this point it would be fairer to say that we simply don't know. It will not take away anything from the hypothesis.

### Author's response

*Although we respect the reviewer's point of view, we feel that the discussion about the origin of mitochondria and phagocytosis is important. In fact it has been a central focus of debate in the literature of eukaryogenesis. For the reasons already presented in our reply to Anthony Poole, we favor the view that a primitive phagocytic machinery cannot be ascribed to the incipient phase of eukaryogenesis*.

The literature on the role of oxygen in eukaryote evolution and compartmentalization should be covered more extensively. Important papers on the subject are not mentioned including Nature 445,47 (2006) or BBRC 363, 885 (2007).

### Author's response

*We now include the citation to the BBRC paper of 2007. The hypothesis raised by the Nature 445,47 (2006) paper remains controversial (see MBE, 2008 25(9):1931-1942), therefore we did not use it to support our model*.

Other hypotheses about the driving forces behind the origin of the nucleus should be mentioned, including: Nature 440, 41 (2006); Biol Direct 3, 31 (2008); Biol Direct 5, 7 (2010). It should also be pointed out that a role for biofilms and syntrophy in the origin of eukaryotes has already been proposed: J Mol Evol 47, 517 (1998); Nat Rev Genet 8, 395 (2007); Biol Direct 2, 3 (2007).

### Author's response

*We included the missing citations in the revised version of the manuscript*.
